# First record of the genus *Pycnodictya* with its subspecies *P. galinieri galinieri* from Egypt (Orthoptera, Acrididae)

**DOI:** 10.3897/zookeys.630.10162

**Published:** 2016-11-09

**Authors:** Asmaa A. Haggag

**Affiliations:** 1 Entomology Department, Faculty of Science, Cairo University, Giza, Egypt

**Keywords:** Egypt, new record, Oedipodinae, Pycnodictya galinieri

## Abstract

The band-winged *Pycnodictya galinieri galinieri* (Reiche & Fairmaire, 1849) and its genus *Pycnodictya* Stål, 1873 (Orthoptera: Acrididae: Oedipodinae) are recorded for the first time for the Egyptian fauna. The species was collected from Gabal Elba, in the southeastern corner of Egypt. This record expands the known distributional range of *Pycnodictya galinieri* towards the north of Africa. Descriptions of the genus and the Egyptian subspecies are given using multiple diagnostic characters. The descriptions are supplemented by drawings and photographs of the specimen collected. It is proposed that the genus *Pycnodictya* belongs to the tribe Locustini.

## Introduction

The genus *Pycnodictya* Stål, 1873 is a member of the subfamily Oedipodinae. At present it includes 14 species, one of which contains two subspecies (Eades et al. 2016), mainly distributed over the Afrotropical region (Johnston 1956, Dirsh 1965), with three species (*Pycnodictya dentata* Krauss, 1902; *Pycnodictya galienieri* Reiche & Fairmaire, 1849; *Pycnodictya gracilis* Uvarov, 1936) reaching the Arabian Peninsula (Popov 1980, Ingrisch 1999, Eades et al. 2016).

*Pycnodictya* is arguably one of the rarer but morphologically distinct genera in the Oedipodinae. However, its species are not easily distinguished morphologically as many previous descriptions considered the color of hind wing and leg as main diagnostic characters (Uvarov 1929). *Pycnodictya* is unique in having an expanded lower marginal area of the hind femur, by which it can be easily distinguished from related genera of Oedipodinae, such as *Chloebora* and *Scintharista* described by Saussure in 1884 (Dirsh 1965). Generally most Oedipodinae have brightly colored hind wings, are characterized by the absence of a prosternal process, the hind legs are missing an external apical spine at the knee and stridulatory serration on the inner surface of hind femur, and by presence of an intercalary vein in medial area of tegmina and the vertical frons (Bolívar 1876, Siddiqui and Shamim 2013).

Previously, the subfamily Oedipodinae was represented in Egypt by 44 species and subspecies, belonging to five tribes: Acrotylini, Epacromiini, Locustini, Oedipodini, and Sphingonotini (Abdel-Dayem et al. 2005, Haggag et al. 2008, Haggag 2011) The genus *Pycnodictya* is currently listed under Oedipodinae without assignment to any of the tribes (Eades et al. 2016); in this article, the tribe Locustini is proposed for this genus following Johnston (1956), who used tribe names as group names, and included *Pycnodictya* in the group Locustae (valid tribe Locustini).

*Pycnodictya* was established by Stål (1873) to include *Pycnodictya obscura* Linnaeus, 1758 and *Pycnodictya rosacea* Serville, 1838, but the latter has been recently considered a synonym of the first (Eades et al. 2016). Some species of this genus are only known by only one of the two sexes and descriptions are often based upon a single specimen, as for *Pycnodictya citripennis* Saussure, 1888; *Pycnodictya dentata* Krauss, 1902; *Pycnodictya herero* Karny, 1910; and *Pycnodictya kelleri* Schulthess, 1894. Thus a revision of the genus including a key to the species is necessary.

Sporadic faunistic investigations had been made to record Egyptian insect species in different regions of the country. Some information about the insect fauna of Gabal Elba was reported by Hassan and Fadl (2000); however, they did not record *Pycnodictya galinieri* (Reiche & Fairmaire, 1849).

Gabal Elba covers approximately 10,000 km^2^, includes a group of six mountains, and is considered a distinct phytogeographic region of Egypt (Al-Gohary 2008). Moreover, it is considered to be a transitional zone between the Afrotropical and the Palaearctic biogeographical regions with a special ecogeographical area located in Egypt that lead to its declaration as a natural protectorate in 1984 (Hassan and Fadl 2000).

This contribution is the first record for the Afrotropical genus *Pycnodictya* Stål from Egypt. Based on microscopic observations of external morphological characters and a comparison to earlier literature dealing with the description of different species of this genus, especially from East Africa and Yemen (Ingrisch 1999), the available band winged specimen was identified as *Pycnodictya galinieri galinieri* (Reiche & Fairmaire), which was described originally from Ethiopia and has been recorded further from Eritrea, Kenya, Oman, Somalia, South Africa, Sudan, Tanzania and Yemen (Johnston 1956, 1968; Dirsh 1965; Popov 1980; Ingrisch 1999; Eades et al. 2016).

## Material and methods

The specimen was collected from Haliab, during an extensive survey by Egyptian taxonomists to the natural protectorate of Gabal Elba, who brought it with other orthopteriod specimens to me to identify as specialist in Egyptian Orthoptera, and then it was dry mounted. The terminology of morphological characters used here is adopted from Chopard (1943) and Dirsh (1965). Morphological features were measured with an ocular micrometer and drawn with aid of a *camera lucida* attached to a Hund Wetzlar SM33 stereomicroscope. Drawings and photographs were modified with Adobe Photoshop C5 software and the distributional map was produced with Arc View 3.2. Photographs of the species were taken by a Nikon D5300 digital camera, Af-S zoom, Nikkor 18–55 mm 1:3.5–5.6 GII.

The measurements are in millimeters and the whole length of the specimen was measured along the midline from fastigium of the vertex to the distal end of the external genitalia, that of hind femur was measured from the basal to the most apical point, and the tegminal length was measured laterally along its greatest length.

## Taxonomy

### Family Acrididae Subfamily Oedipodinae Tribe Locustini

#### Genus Pycnodictya

Taxon classificationAnimaliaOrthopteraAcrididae

Stål, 1873

Pycnodictya Stål, 1873: 116, 121.

##### Type-species.

*Gryllus obscurus* Linnaeus, 1758: 433.

##### Diagnosis

(adapted from Stål 1873, Saussure 1884, Dirsh 1965). Species belonging to this robust genus are well recognized by their thick hind femur having the upper carina serrated and the lower marginal area highly expanded, as well as by their robust, rugose head and pronotum, and their brightly colored hind wings with dark or at least smoky transverse fascia. They are also generally characterized by their globular heads with prominent eyes and filiform antennae, a distinct median carina of the pronotum crossed by the third transverse sulcus that continues on the lateral lobes, while the lateral carinae are absent and the posterior margin is acutely angular; the meso- and metasternal interspace are very wide. The elongate supra-anal plate is angular, the cerci are narrowly conical with subacute apices, and the subgenital plate is conical with an obtuse apex in males. The female ovipositor valves are short, robust with curved apices.

#### Pycnodictya galinieri galinieri

Taxon classificationAnimaliaOrthopteraAcrididae

(Reiche & Fairmaire, 1849)

Figs 1–8 , 9–14

Oedipoda galinieri Reiche & Fairmaire, 1849: 432.
Humbe hyalodes Karsch, 1896: 265.
Humbe miniatipennis Karsch, 1896: 265.

##### Type specimen.

Unspecified male collected from Ethiopia deposited in Muséum national d’Histoire naturelle Paris, France (MNHN).

##### Material examined.

1♀, Halaib II in Gabal Elba (22°11'16"N, 36°22'14"E), 2003 [CUE, Efflatoun Bey Collection, Entomology Department, faculty of science, Cairo University, Egypt].

##### Description.

**Figures 1–8.**
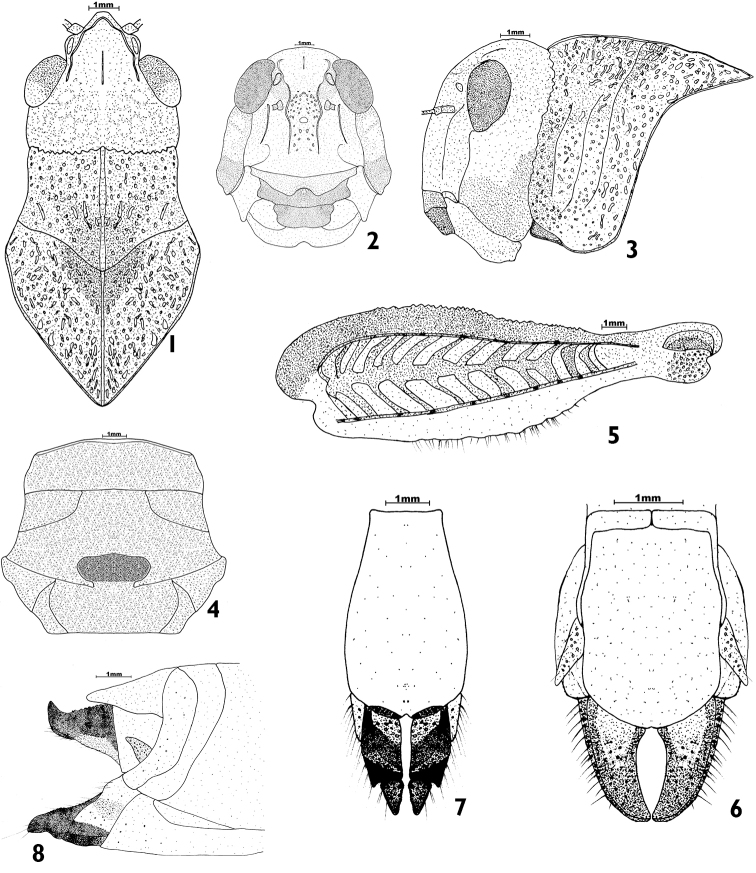
♀ *Pycnodictya galinieri galinieri*. **1** dorsal view of head and pronotum **2** lateral view of head and pronotum **3** anterior view of head **4** ventral view of meso- and metasternum **5** external side of hind femur **6** dorsal view of abdominal apex **7** ventral view of abdominal apex **8** lateral view of abdominal apex.

**Figures 9–14.**
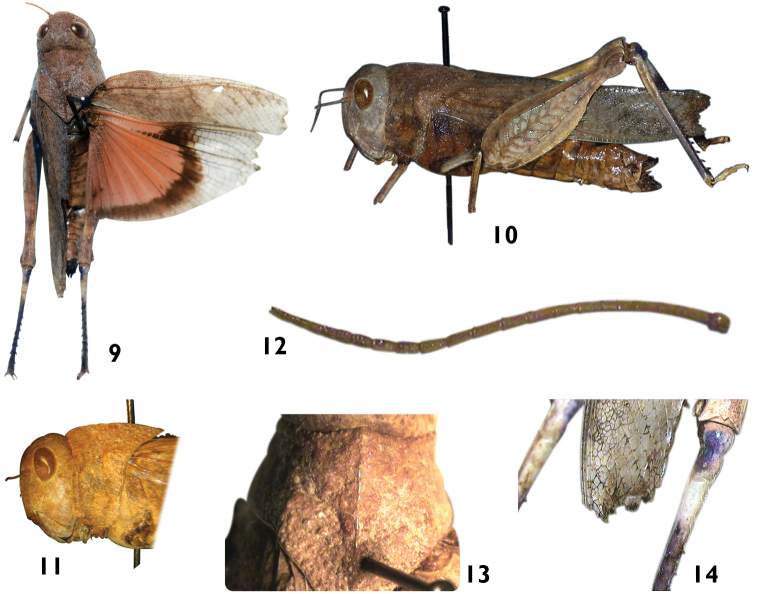
Digital photos of female *Pycnodictya galinieri galinieri* **9** dorsal view **10** lateral view **11** lateral view of head & pronotum **12** antenna **13** dorsal view of pronotum **14** dorsal view of base of hind tibia.

The body of the female is robust, medium-sized, and brownish, with sparse hairs on pronotum, sternum, legs, and mouth parts. Head (Figs 1, 9) rugose, prominent, and straight. Eyes rounded with obtuse rounded apex. Frons (Fig. 3) with rounded obtuse angle to fastigium of vertex. Frontal ridge shallowly sulcate with obtuse lateral carinulae, wide above ocellus, excurved between antennae then straight below ocellus, not reaching clypeus; surface coarsely punctured and wrinkled above ocellus. Fastigial foveolae shallow and oval. Fastigium of vertex wide, shallow with obtuse margins. Vertex broad and convex with low carinula between eyes. Antennae (Fig. 12) yellowish brown, filiform, with 27 flagellomers, shorter than head and pronotum together.

*Pronotum* (Figs 1, 9, 13) constricted in the posterior half of prozona, coarsely punctured and wrinkled especially in metazona; anterior margin dentate with slightly acute angle at median carina; third transverse sulcus sharp; metazona coarsely wrinkled with tubercles, its length slightly longer than prozona, posterior angle highly acute angular, median carina obtuse, distinct, crossed by third transverse sulcus only and raised in prozona. Lateral lobes (Figs 2, 10, 11) with three transverse sulci, with anterior and posterior margin straight, anterior and posterior lower angle obtusely rounded and lower margin distinctly convex from second sulcus to posterior margin. Mesosternal interspace (Fig. 4) about three times as broad as long and metasternal interspace about 3.25 times as broad as long.

*Elytra* (Fig. 9) wide, about 4.25 times as long as its maximum width, slightly narrowing toward obliquely truncate apex; opaque and with obtuse dark spots that do not form definite transverse bands, leaving the apical third transparent with brownish veins; second branch of medial vein with five branches apically; intercalary vein straight then raised apically, behind middle closer to cubital vein.

*Wings* (Fig. 9) approximately twice as long as its maximum width, with orange red basal half, surrounded by a dark, moderately narrow, transverse semicircular band that does not reach posterior margin, with short anterior projection toward base; veins darkened in transparent apical part.

*Hind femora* (Figs 5, 10) thick, their lengths approx. 3.25 times their maximum widths; upper margins distinctly serrate and lower marginal areas expanded with irregular edges; upper and lower external carinulae with dark dots; inner sides blackish below upper carina and with dark crest at knee.

*Hind tibiae* (Fig. 14) blackish violet except for yellowish ring in basal third and blackish violet condyle internally; shorter than femora with ten spines on outer, eleven on inner side.

*Abdominal extremity* (Figs 6, 7, 8) with ovipositor valves robust, short with curved apex.

The male is noted to be similar to the female but smaller in size; hind wings bright orange red; hind tibiae with a less distinct pale basal ring (Ingrisch 1999).

##### Measurements.

(Table 1).

**Table 1.** Measurements (in mm) of female *Pycnodictya galinieri galinieri* from Egypt (male after Saussure 1884).

Sex	Body	Pronotum	Elytron	Wing	Hind femur	Hind tibia
♀ (mm)	33	9	28	27	17.5	15
♂ (mm)	25	7	26	-	16	-

##### Distribution.

**Figure 15.**
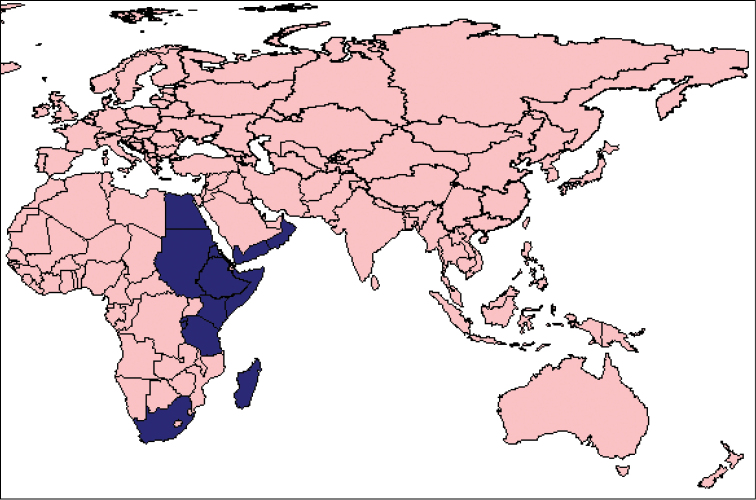
Map of the known country records of *Pycnodictya galinieri galinieri*.

**Figure 16.**
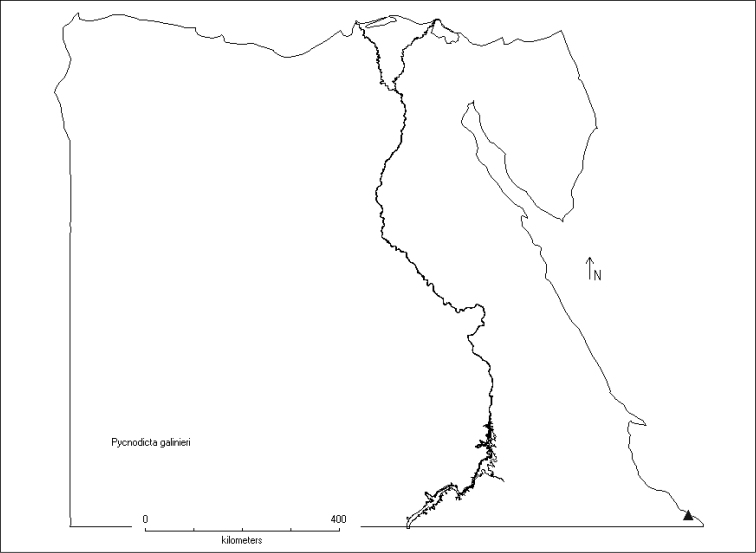
Map of Egypt showing the local distribution of *Pycnodictya galinieri galinieri*.

Afrotropical species distributed along the Indian Ocean in the eastern half of the African continent from Sudan in the north to South Africa (Johnston 1956, 1968; Dirsh 1965; Eades et al. 2016), expanding north eastwards to the south of the Arabian Peninsula to Yemen (Ingrisch 1999) and Oman (Popov 1980), and reaching, with the new record presented here, the southern corner of Egypt at the Red Sea (Figs 15, 16).

## Discussion

The classification of Egyptian Oedipodinae species was previously reviewed by Abdel-Dayem et al. (2005) and Haggag et al. (2008), and tribe Epacromiini was revised under Acridinae by Haggag (2011). The genus *Pycnodictya* belongs to the subfamily Oedipodinae with its brightly colored hind wings, vertical frons, and by the presence of an intercalary vein in medial area of fore wings. Also it lacks the prosternal process of other Acridid subfamilies (e.g. Calliptaminae, Cyrtacanthacridinae and Eyprepocnemidinae) and stridulatory serration on the inner surface of hind femur of other subfamilies (e.g. Gomphocerinae and Eremogryllinae).The genus *Pycnodictya* is recorded here as its subspecies *Pycnodictya galinieri galinieri* and new for the Egyptian fauna from Gabal Elba.

The expanded lower marginal area of the hind femur is very characteristic for *Pycnodictya* by which it can easily be separated from related genera such as *Chloebora* Saussure, 1884 and *Scintharista* Saussure, 1884 (Dirsh 1965).

The different species of the genus *Pycnodictya* are similar to one another in general appearance, and the most features used for their identification are the color of the hind wings and hind legs, which are easily viewed by eye (Uvarov 1929, Ingrisch 1999). The previous description of *Pycnodictya galinieri* by Saussure (1884) is superannuated and insufficient but recognizable, and thus, a description of the Egyptian subspecies is given in this paper with additional line drawings and photographs that are not available elsewhere. *Pycnodictya galinieri galinieri* is well-differentiated from other species of the genus by its characteristic hind wings with orange red bases, moderately narrow dark band with an anterior projection that is separated from the posterior margin, and the clearly dentate anterior margin of the pronotum. In addition, the hind tibia is blackish violet with a yellowish basal ring and the hind femur is blackish on its inner side.

*Pycnodictya galinieri* has a dentate anterior margin of pronotum resembling the situation in *Pycnodictya dentata* Krauss, 1902 but lacks the sinuated posterior lower angle of the pronotal lateral lobes of the latter. However, *Pycnodictya kelleri* Schulthess and the other subspecies *Pycnodictya galinieri citrina* Kevan, 1961 described from Somalia and restricted to it (Eades et al. 2016), differentiated well from *Pycnodictya galinieri galinieri* with their sulphorous or yellowish wings, respectively. Only the two species *Pycnodictya diluta* Ramme, 1929 and *Pycnodictya zinae* Uvarov, 1949 resemble *Pycnodictya galinieri* in the purplish hind tibia, but *Pycnodictya diluta* with disappeared fascia of the hind wings and *Pycnodictya zinae* with crested pronotal median carina. Moreover, *Pycnodictya gracilis* Uvarov and *Pycnodictya kilosana* Miller, 1929 distinguished from *Pycnodictya galinieri* as their hind tibia is honey yellow or light brown not blackish violet as the latter.

*Pycnodictya galinieri* is widespread along the eastern part of the African continent. The high mobility of Oedipodinae as very strong fliers (Alexander 1964) may explain the wide distribution of *Pycnodictya galinieri* from South Africa to Egypt. It is not clear, whether the subspecies has so far been overlooked in Egypt or whether it has expanded its range. The new finding and previous new records from Egypt (Haggag et al. 2008, Haggag 2011) highlight the importance of making a thorough survey of Acridoidea in diverse regions of the country.

## Supplementary Material

XML Treatment for Pycnodictya

XML Treatment for Pycnodictya galinieri galinieri
